# The Effect of Erosive Media on the Mechanical Properties of CAD/CAM Composite Materials

**DOI:** 10.3390/jfb15100292

**Published:** 2024-10-01

**Authors:** Marwa M. Alnsour, Rasha A. Alamoush, Nikolaos Silikas, Julian D. Satterthwaite

**Affiliations:** 1 Department of Restorative Dentistry, School of Dentistry, University of Jordan, Amman 11942, Jordan; 2 Dental Department, Jordan University Hospital, Amman 13046, Jordan; 3 Department of Fixed and Removable Prosthodontics, School of Dentistry, University of Jordan, Amman 11942, Jordan; 4 Division of Dentistry, School of Medical Sciences, University of Manchester, Manchester M13 9PL, UKjulian.satterthwaite@manchester.ac.uk (J.D.S.)

**Keywords:** PICN, polymer-infiltrated ceramic-network, RCB, resin composite blocks, acidic media, microhardness, flexural strength, elastic modulus

## Abstract

This study aimed to investigate the effect of acidic media storage (gastric acid and Coca-Cola) on the mechanical properties of CAD/CAM materials. Three types of materials were tested: a polymer-infiltrated ceramic network (PICN) (Vita Enamic (En), VITA Zahnfabrik, Germany), a resin composite block (RCB) (Cerasmart (Cs), GC Corp, Japan), and a conventional resin-based composite (Gradia direct (Gr), GC Corp, Japan), which was used as a control. Beam-shaped specimens of each material, with dimensions of 16 mm × 4 mm × 1.5 mm, were prepared (90 in total). The specimens were divided into subgroups (10 each) and stored for 96 h in either gastric acid, Coca-Cola, or distilled water. Flexural strength and elastic modulus were evaluated using a three-point flexural strength test with acoustic emission (AE) monitoring. Vickers microhardness was measured before and after storage in gastric acid and Coca-Cola. Data were statistically analysed using two-way and one-way ANOVA, the Tukey’s post hoc, and independent *t*-test at a significance level of 0.05. The results showed that Cs and En maintained their flexural strength and elastic modulus after acidic media exposure, while Gr experienced a significant decrease in flexural strength following gastric acid storage (*p* < 0.01). Initial crack detection was not possible using the AE system, impacting the determination of flexural strength. Exposure to acidic media decreased all materials’ microhardness, with Gr showing the most notable reduction (*p* < 0.0001). Gastric acid had a greater impact on the microhardness of all tested materials compared to Coca-Cola (*p* < 0.0001). In conclusion, storage in erosive media did not notably affect the flexural strength or elastic modulus of CAD/CAM composites but it did affect hardness. CAD/CAM composite blocks demonstrated superior mechanical properties compared to the conventional composite.

## 1. Introduction

Dental erosion, characterized by the progressive loss of hard tooth tissue exposed to non-bacterial acids, presents a significant concern in the dental practice [1]. Dental erosion can be caused by extrinsic or intrinsic factors. Extrinsic erosion often results from the prolonged consumption of acid-containing food and beverages such as carbonated soft drinks, citrus fruits, and alcoholic beverages, while intrinsic erosion occurs when gastric acid enters the oral cavity, which is commonly observed in individuals with conditions like gastroesophageal reflux disease (GERD) and psychological eating disorders [2].

The prevalence of dental erosion varies widely across populations, and it is influenced by socioeconomic factors, dietary habits, and overall health status [3]. Prevalence studies have revealed a concerning trend of increasing erosion-related tooth wear, particularly among younger populations [2]. Clinically, erosion manifests as a loss of enamel and dentine, which is accompanied by the cupping and shortening of the tooth surface, leading to both aesthetic and functional impairments. However, patients often seek intervention only after substantial tooth damage which necessitates restorative intervention [1,2].

The restoration of eroded teeth depends on the extent of tooth loss, ranging from simple restorations to full mouth rehabilitation [1,2]. Historically, conventional materials such as cast gold or metallo-ceramic restorations have been used to treat dental erosion. Innovations in dental materials have broadened treatment options to include more aesthetic materials, including resin composites, glass ceramics, and polycrystalline zirconia [4]. Although glass ceramics and zirconia have superior mechanical properties and aesthetics, resin composites, including conventional and CAD/CAM composites, are gaining attention. This could be attributed to their reparability and conservative nature, particularly in younger patients with dental erosion, thus slowing the restorative cycle [4,5]. However, concerns persist regarding the perceived weakness and the higher maintenance of composites compared to ceramics [6].

While conventional composites are the most used resin-based materials in dental practice, CAD/CAM resin-based composite blocks, shortly CAD/CAM composite blocks, are increasingly recognized as a viable restorative option. CAD/CAM composite blocks encompass two main types: resin composite blocks (RCBs) and the polymer-infiltrated ceramic-network (PICN). The primary distinction between CAD/CAM composite blocks and conventional composites lies in their composition and manufacturing processes. Both conventional composites and RCBs consist of a polymeric matrix reinforced by fillers. While conventional composites require chairside curing, RCBs are pre-polymerized under high temperature and pressure into ready-to-mill blocks [7]. Studies have shown that high-temperature and high-pressure (HT/HP) polymerization can enhance the mechanical properties of RCBs [8]. On the other hand, the polymer-infiltrated ceramic network (PICN) has emerged as a promising option in restorative dentistry. The PICN consists of a ceramic network that is infiltrated by resin polymer, combining the advantages of resin composites and glass ceramics, and offering the high strength of ceramics along with the elasticity and ease of repair of composites [6].

In the context of stability in acidic media, conventional composites have shown structural changes and deterioration after acidic exposure [9,10]. On the other hand, RCBs have shown stability during short-term acidic exposure [11,12,13,14,15,16]. Despite manufacturers’ claims of superior properties, the true performance of PICN, especially in response to erosive fluids, is not completely understood and needs further investigation.

A limited number of studies have investigated the impact of erosive agents on PICN CAD/CAM blocks, particularly gastric acid, and Coca-Cola over a short exposure duration of less than 24 h, concluding no significant effects of such erosive agents on the flexural strength and microhardness of the PICN block [11,12,13,17,18]. This contrasts with other studies that have reported a reduction in flexural strength and microhardness after storing PICN blocks for longer periods, using different acids [19,20]. Thus, there is a need to reassess the effects of gastric acid and Coca-Cola on PICN blocks over extended durations. To the best of the authors’ knowledge, no studies have evaluated the flexural strength and microhardness of PICN blocks exposed to gastric acid and Coca-Cola for durations longer than 24 h.

Moreover, existing studies assessing the strength of PICN blocks have primarily reported ultimate strength values, which often overestimate the material’s strength. Dental restorations commonly fracture at stresses well below their ultimate strength due to fatigue [21]. To address this issue, acoustic emission (AE) technology presents a viable solution [22]. When materials develop cracks, they emit high-stress energy waves known as acoustic emission throughout the process from initial crack formation until reaching the failure point. Therefore, coupling an appropriate AE system with a mechanical testing machine could enable the detection of the initial crack point and obtain values for flexural strength that are more reliable.

Therefore, our study aims to not only evaluate the performance of PICN blocks compared to RCB and conventional direct composite under longer storage time in gastric acid and Coca-Cola but also to utilize acoustic emission technology to enhance our understanding of their fracture behavior. By integrating this advanced analytical technique, we can have a more reliable measurement of these materials’ strength. There are three null hypotheses for this study. (1) There is no significant difference in the flexural strength and elastic modulus of PICN blocks compared to both RCBs and conventional direct composites when exposed to gastric acid and Coca-Cola. (2) There is no significant effect of exposure to gastric acid and Coca-Cola on the flexural strength and elastic modulus of the investigated materials. (3) There is no significant difference in microhardness within and between the investigated materials before and after their exposure to simulated gastric acid and Coca-Cola.

## 2. Methodology

### 2.1. Specimen Preparation

Three materials were investigated: a polymer-infiltrated ceramic-network (PICN) block (Vita Enamic (En), VITA Zahnfabrik, Bad Sackingen, Germany), a resin composite block (Cerasmart (Cs), GC Corp, Bunkyo City, Japan), and a conventional direct composite (Gradia Direct (Gr), GC Corp, Bunkyo City, Japan) (Table 1). Following the ISO standards 6872:2015 + A1:2018, ninety specimens were prepared comprising 30 specimens of each material [23]. Specimens were prepared into bars with rectangular cross-sections and chamfered edges of 4 mm width (w), 1.5 mm thickness (b), and 16 mm length (l). PICN and RCB were sectioned using a high-speed precision cutting machine SOMET 1000 Precision Saw (Buehler Ltd., Lake Bluff, IL, USA) with a metal-bonded diamond cut-off wheel (M0D13) at 3800 rpm and 0.05 mm/s feed speed under water cooling.

Conventional direct composite specimens were prepared using a bar-shaped polytetrafluoroethylene (PTFE) mold in which composite was pressed and cured using Elipar TM S10 LED Curing Light (3M ESPE, St. Paul, MN, USA) five times on the top and bottom surfaces for 20 s each. Subsequently, all bars were polished with metallographic silicon carbide paper (sequence of 600, 800, 1000, 1200-grit) and underwent ultrasonic cleaning in distilled water for 10 min. The final dimensions of all specimens after polishing were 16 mm × 4 mm × 1.5 mm, (± 0.1) and were measured using a digital caliper.

The specimens of each material were then randomly allocated into three groups: Group 1: control group; the specimens were stored in distilled water (n = 10); Group 2: the specimens were stored in gastric acid (n = 10) (enzyme-free simulated gastric acid consists of 1.9–2.1 g/l sodium chloride (pH 1.15–1.25, at 25 °C at 26× dilution) (J.T. Baker, Phillipsburg, NJ, USA)), and Group 3: the specimens were stored in Coca-Cola (n = 10). The specimens were individually immersed in 3 mL of the respective media for 96 h in sealed containers. The pH values of the media were measured using a SciQuip Standard Benchtop pH meter (SciQuip, Wem, Shropshire, UK). The pH was 1.2 for gastric acid, 2.5 for Coca-Cola, and 6.9 for distilled water.

### 2.2. Three-Point Flexural Strength Test, Elastic Modulus, and Acoustic Emission Analysis

The flexural strength and elastic modulus were determined using a Universal Testing Machine (Zwick Roell Z020, 0.5 kN X force HP load cell, Ulm, Germany) with a crosshead speed of 0.5 mm/min and a support span of 14 mm. The loading scheme employed a three-point loading setup (ISO standards 6872:2015 + A1:2018), where the load roller was in the middle of the support span = 7 mm with a loading span to support span ratio of ½ [23]. The specimens were loaded until a fracture occurred. Simultaneously, an Acoustic Emission (AE) system (1283 USB AE node, Physical Acoustics Corporation, West Windsor, NJ, USA) was employed to detect audible signals produced during the loading process. The AE sensor (PK61, Physical Acoustics Corporation, West Windsor, NJ, USA) was positioned on the support roller ≈ 2 mm below the specimens, and wave collection began simultaneously with the loading process.

Graphs and data from the Zwick Roell (TestXpert^®^ V12.6) and AEwin™ (E5.32). software, loading force versus time and the amplitude of the acoustic waves versus time, were compared using the x-axis (time) as a reference. A predetermined 45 dB amplitude threshold was selected (to avoid noise signals from surroundings) as a reference with waves above indicating the crack initiation. The load value, at which the first acoustic amplitude ≥ 45 was recorded, was used to measure the initial flexural strength of the material, whereas the load value, at which the specimens were fractured, was used to measure the final flexural strength of the material using Equations (1) and (2):

σ_1_ = (3F_1_L)/(2bh^2^) (1)

σ_2_ = (3F_2_L)/(2bh^2^) (2)

where (σ_1_) is the flexural strength once the specimens developed a crack in MPa, (σ_2_) is the flexural strength when the specimen fractured in MPa, (F_1_) is the load at which acoustic signals ≥ 45 dB were first recorded in Newtons, (F_2_) is the load at fracture in Newtons, (L) is the length of specimen in mm, (b) is the width at the center of the specimen in mm, and (h) is the height at the center of the specimen in mm.

The elastic modulus was calculated using Equation (3):

E = (Fl^3^)/(4bh^3^ d) (3)

where d is the deflection, in millimeters, at load F, l is the distance between the support pens, in millimeters, and b and h are as defined above.

### 2.3. Microhardness

Microhardness was measured for 10 specimens of each material (5 stored in gastric acid and 5 in Coca-Cola), totaling 30 specimens. Measurements were taken before and after storage using a microhardness tester (FM-700, Future Tech Corp, Tokyo, Japan).with a Vickers indenter at 1000 gf load for 15 s [13]. Five indentations were made on each specimen (0.5 mm apart) before and after storage in the gastric acid and Coca-Cola for 96 h. The diagonal lengths (d1 and d2) of the indentation were measured, and equation (4) was used to calculate the microhardness.

VHN = FA ≈ 1.8544F/d^2^ (4)

where F is the applied force in kilograms and A is the indentation surface area in millimeters squared.

### 2.4. Morphological Analysis

A scanning electron microscope (Tescan, VEGA3 VP-SEM, Brno, Czech Republic) was utilized to examine one specimen from each material group before and after acidic storage. Each specimen was affixed to aluminum stubs (12 mm thickness) using double-sided adhesive tabs and sputter-coated with silver. Scanning electron micrographs were captured at a magnification of 1.00 kx, accelerating voltage of 5 kV, spot size of 3.5, and working distance (WD) of 14 mm focusing on representative areas of the samples.

### 2.5. Statistical Analysis

The data were analyzed using IBM SPSS Statistics version 25.0.0.2 (SPSS Inc., Chicago, IL, USA). Tests for normality (Kolmogorov–Smirnov test) and equal variance (Levene’s test) were performed. To evaluate the flexural strength, elastic modulus, and microhardness, a two-way analysis of variance (ANOVA) was conducted to examine the effects of material type and storage media. Subsequently, one-way ANOVA followed by Tukey’s post hoc analysis was employed for multiple comparisons among different materials and storage media conditions. In addition, an independent *t*-test was performed to compare the microhardness of same material before and after storage in different media. The level of significance was set at α = 0.05.

## 3. Results

### 3.1. Flexural Strength and Acoustic Emission

Two-way ANOVA showed a significant material effect and insignificant effect of storage media with a significant interaction (*p* < 0.0085) in which the material effect, the simple main effect (0.95), had more influence on the flexural strength than the storage media effect (0.0003).

One-way ANOVA followed by Tukey’s post hoc analysis was performed for multiple comparisons between different materials and different storage media (Table 2), which revealed a significant difference between materials (*p* < 0.0001). Specifically, Cs exhibited the highest flexural strength (236.4 ± 20.4 MPa), followed by En (154.5 ± 15.7 MPa), with Gr (93.7 ± 12.5 MPa) displaying the lowest flexural strength.

Within the same material groups, no significant change was observed in the flexural strength of Cs and En after exposure to gastric acid and Coca-Cola. However, Gradia experienced a decrease in flexural strength after exposure to both gastric acid and Coca-Cola (78.7 ± 8.6 MPa and 86.3 ± 5.9 MPa, respectively) with the difference being statistically significant only after exposure to gastric acid (*p* < 0.01).

**Figure 1:**
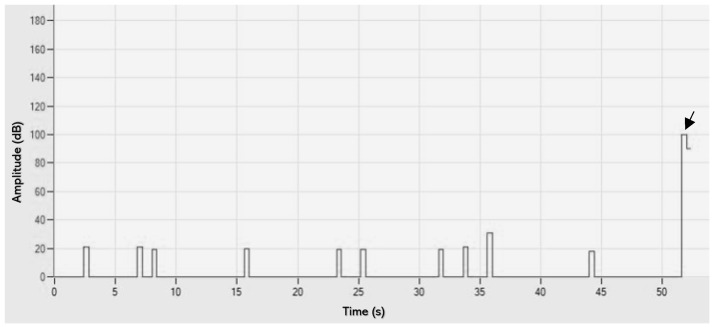
Printed screen from AEwin software. The Y-axis represents the amplitude (dB unit) that has been recorded by the acoustic emission machine during the loading process while the X-axis represents time (Seconds). The only amplitude peak that exceeds the 45 dB threshold is the one at the fracture point (see the arrow).

The acoustic emission (AE) setup that has been used in the present study was incapable of detecting initial cracks. Energy release has only been detected at the failure point for each material. The only exception was three specimens of En. Overall, no initial crack has been detected and it was not possible to calculate the initial flexural strength. Consequently, no data are available to compare the initial and ultimate flexural strength (Figure 1).

### 3.2. Elastic Modulus

A two-way ANOVA showed significant material (*p* < 0.0001), and storage media (*p* < 0.02) effects with an insignificant interaction in which the material effect, the simple main effect (0.98), had more influence) on the elastic modulus than the storage media effect (0.0013).

One-way ANOVA followed by Tukey’s post hoc analysis was performed for multiple comparisons between different materials and different storage media which revealed a significant difference between materials (*p* < 0.0001). Specifically, En exhibited the highest elastic modulus value (28.1 ± 1.2 GPa), followed by Cs (10.3 ± 0.7 GPa), with Gr (7.0 ± 0.8 GPa) displaying the lowest elastic modulus (Table 3). Within the same material groups, no significant change was observed in the elastic modulus after exposure to gastric acid and Coca-Cola.

### 3.3. Microhardness and Scanning Electron Microscope

Two-way ANOVA showed significant material (*p* < 0.0001) and storage media effects with a significant interaction (*p* < 0.0001) on microhardness, in which the material effect, the simple main effect (0.72), had more influence than the storage media effect (0.15).

Before storage in acid, the hardness of the materials was as follows: En (228.9 ± 0.8), Cs (87.9 ± 1.2), and Gr (40.8 ± 0.4) (*p* < 0.0001) (Table 4). The storage in gastric acid and Coca-Cola has decreased the materials’ microhardness without altering this order (*p* < 0.0001). Hardness reduction after storage in gastric acid was highest for Gr with a 30.6% reduction followed by En at 10.3% and then Cs at 8.6%. Similarly, hardness reduction after storage in Coca-Cola was highest for Gr with 16% reduction but it resulted in a higher reduction in hardness for Cs than for En with 7.3% and 6.3% respectively. Moreover, storage in gastric acid has resulted in higher hardness reduction than storage in Coca-Cola for all of the investigated materials (*p* < 0.0001).

The SEM images of all examined materials showed an increase in surface irregularities after storage in gastric acid and Coca-Cola. This increase was more obvious for Gr and En than for Cs. Bubbles were observed on Gr and Cs specimens that were stored in Coca-Cola. All materials have shown a loss of polymer content after storage in gastric acid and Coca-Cola, whereas only Gr showed filler loss.

## 4. Discussion

There were significant differences in flexural strength and elastic modulus between conventional direct composite and CAD/CAM composite blocks before and after their exposure to gastric acid and Coca-Cola, and thus the first null hypothesis has been rejected. In this study, Cs demonstrated higher flexural strength than En, and both Cs and En showed higher flexural strength than Gr. These findings align with previous research [11,24,25,26] indicating that Cs generally demonstrates superior flexural strength compared to En, and CAD/CAM composites exhibit higher flexural strength than conventional composites.

This can be explained by the higher non-resin content of En (feldspathic ceramic skeleton) compared to Gr’s non-resin content (silica filler) (86% vs. 76%, respectively). However, Cs has a filler content slightly less than that of Gr (71% vs. 76% respectively), which indicates that other factors like filler size and polymerization technique are involved.

Gr composite is polymerized using a light cure unit, whereas the resin component of En and Cs are polymerized under heat and pressure besides the light cure unit. The application of heat and pressure results in a higher degree of conversion of the resin and more crosslinking, giving a stronger material [8]. Regarding the filler size, Cs consists of a mixture of silica and barium glass fillers in both nanoscale and microscale levels, where nanofillers exhibit greater strength than the microscale non-resin components (feldspathic and silica) found in En and Gr, respectively [27]. The higher flexural strength of Cs compared to En could also be attributed to differences in microstructure and configuration. Cs features a highly filled resin matrix, whereas En incorporates a ceramic skeleton infiltrated with resin polymer. The former configuration is likely to distribute energy more elastically compared to the latter, where the brittle ceramic skeleton is the main structure, potentially decreasing the risk of crack formation.

Nevertheless, the fracture behavior of restorative materials is influenced by its elastic modulus as well. An ideal restorative material should have a balance between its strength and elasticity. The strength should be sufficient to withstand the average masticatory forces (both En and Cs have showed sufficient strength) whereas the elastic modulus should closely match that of the tooth structure.

In this context and upon comparison with the literature, the elastic modulus of Cs in this study was slightly lower than that of dentine, whereas for En, its elastic modulus is between the elastic moduli of enamel and dentine [28,29]. These findings are consistent with previous studies reporting elastic modulus values for Cs (7.6–12.1 GPa) and En (21.5–34.5 GPa) [24,25,30,31]. Therefore, the preference for one material over another may depend on the clinical application and whether the restoration will bond to both enamel and dentin or only to dentin.

There was no significant difference in the flexural strength and elastic modulus of the investigated materials, except for Gr, before and after their exposure to gastric acid and Coca-Cola; therefore, the second null hypothesis has been partially accepted.

The flexural strength and elastic modulus of En and Cs were unaffected after 96 h of storage in gastric acid and Coca-Cola. This is consistent with other studies that have reported no difference in the flexural strength of either material after their storage in HCl pH = 1.2 for a maximum time of 24 h [11,17,18]. Although our study extended the exposure to 96 h, the results were similar, indicating that the flexural strength and elastic modulus of the CAD/CAM composite are stable in short-term acid exposure. In contrast, the Gr composite showed a reduction in flexural strength after exposure to both gastric acid and Coca-Cola. This aligns with findings from Scribante et al. (2019), who reported an approximately 8% reduction in strength after storing Gr in Coca-Cola for one week [9]. The difference in response to acidic conditions between Gr and the CAD/CAM composites can be attributed to differences in composition and polymerization techniques.

**Figure 2:**
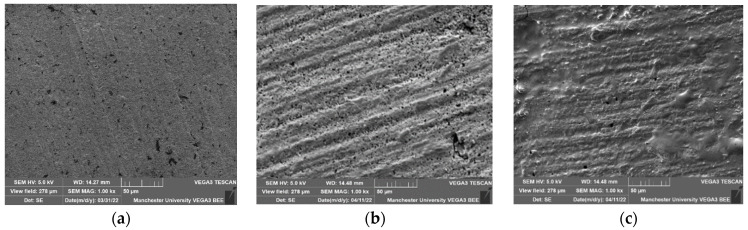

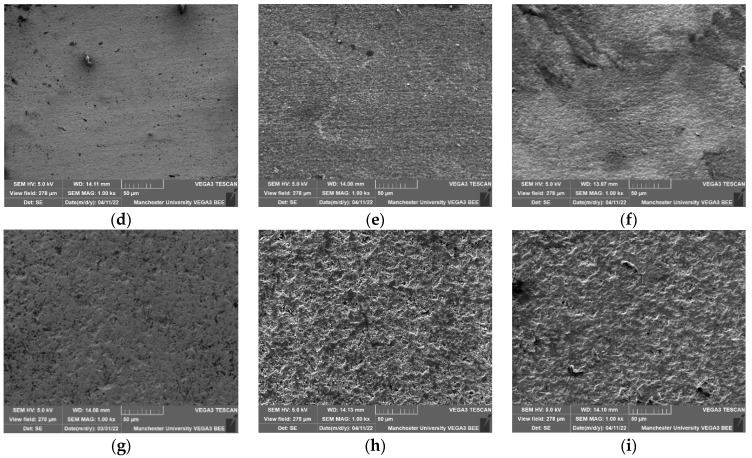
Scanning electron microscope images of the examined materials. (**a**–**c**): Gradia (**a**) without storage in acidic media, (**b**) after storage in gastric acid, and (**c**) after storage in Coca-Cola. (**d**–**f**): Cerasmart (**d**) without storage in acidic media, (**e**) after storage in gastric acid, and (**f**) after storage in Coca-Cola. (**g**–**i**): Enamic (**g**) without storage in acidic media, (**h**) after storage in gastric acid, and (**i**) after storage in Coca-Cola.

Despite the flexural strength and elastic modulus of En and Cs being unaffected by acidic storage, some changes in surface morphology were noted in the SEM images (Figure 2). This could indicate that some degradation is taking place, and a longer storage time may lead to further deterioration and a reduction in strength. A study by Sagsoz and Sagsoz (2019) observed significant changes in the flexural strength of En after seven days of storage in different acids, suggesting that longer exposure periods might reveal more pronounced effects [20].

The null hypotheses that there are no significant differences in microhardness within and between conventional direct composite and CAD/CAM composite blocks before and after their exposure to gastric acid and Coca-Cola have been rejected. En has reported the highest microhardness among the tested materials, and this could be due to its high glass ceramic content (86 Wt% feldspathic). On the other hand, the difference in hardness between Cs and Gr cannot be primarily explained by their filler content or filler size [32]. Rather, the higher microhardness of Cs was mostly due to the difference in the polymerization technique that resulted in a higher degree of conversion of the resin part.

Unlike the elastic modulus and flexural strength, the microhardness of all examined materials has been decreased after their storage in gastric acid and Coca-Cola. This difference is mostly due to surface morphology changes, as confirmed by SEM analysis in our study, rather than structural alterations. Consistent with previous research, Cs and Gr showed decreased hardness after 7–28 days of storage in Coca-Cola. These studies reported reductions of approximately 5% for Cs and 20% for Gr after 7 days, which aligns closely with the reductions observed in our study [14,33]. In a separate investigation, Cs demonstrated a hardness decrease from 77.6 to 72.4 VHN after 232 h in HCl, whereas in our study, Cs hardness decreased from 87.9 to 80.3 VHN after just 96 h in Coca-Cola. Despite the longer exposure duration in their study (2.5 times longer), the reduction in hardness was comparable, suggesting a potential slowing of degradation rates over longer durations. It is worth noting that this reduction was linked to filler leaching from Cs unlike other RCBs in their study. These findings suggest varying effects among RCBs with different chemical compositions.

Conversely, another study reported no change in hardness of En and Cs after 18 h in gastric acid, which is five times less than the storage time in our study [13]. En, however, exhibited a reduced hardness of about 9% after only 24 h in Coca-Cola [34]. Similar findings with different acids showed an En hardness reduction after 7 days [20], which was attributed to polymer degradation and porous ceramic network exposure. This was partially observed in SEM images in our study (Figure 2) and confirmed in the SEM analysis of a previous study [10].

On SEM images, Gr and En showed more pronounced surface irregularities, which might be due to their non-resin components being on a microscale compared to the nanoscale level in Cs [35]. Polymer content loss across all materials highlights the detrimental effects of these acids on both conventional and CAD/CAM composites [10]. The filler loss in Gr suggests less stable filler particles in acidic environments, affecting its structural integrity over time. Our SEM images might suggest that CAD/CAM composites, especially Cs, perform better in resisting surface degradation. However, chemical analysis would be necessary to confirm this hypothesis.

The comparisons above suggest that storage time, acidic media, and composition could potentially influence the mechanical properties of CAD/CAM composite blocks, especially microhardness. Accordingly, one limitation of this study is the use of a limited range of acidic media (gastric acid and Coca-Cola) and CAD/CAM composite blocks (Cr and En). To draw more conclusive results, future studies should explore a broader spectrum of acidic media and include CAD/CAM blocks with diverse compositions. Additionally, conducting multiple readings over extended periods and varying temperatures would enhance the assessment of material durability. Comparing these materials to ceramics as a positive control could further elucidate their relative performance. Furthermore, detailed morphological analyses, such as roughness analysis, could quantitatively and qualitatively assess surface and morphological changes induced by erosive conditions, providing better insight into and explaining the different performances of various materials.

## 5. Conclusions

Based on the limitations and findings of this study, it can be concluded that while storage in acidic media does not significantly affect the flexural strength and elastic modulus of CAD/CAM composite blocks, it does reduce microhardness and alter morphology. These microstructural changes could potentially impact the longevity and clinical performance of these materials. Despite these effects, CAD/CAM composite blocks exhibit superior flexural strength, elastic modulus, and microhardness compared to conventional composites, both before and after storage in acidic media, suggesting a potential for better clinical outcomes. However, clinical studies are necessary to confirm these findings and assess their true clinical performance.

## Figures and Tables

**Table 1:** The manufacturers’ compositional information of the investigated materials. Wt% stands for filler weight percentage.

Product	Material	Composition	Lot. No	Manufacture
Vita Enamic (En)	Polymer-infiltrated ceramic-network CAD/CAM blocks	Feldspathic sintered ceramic network (86 Wt%), infiltrated by urethane dimethacrylate (UDMA) and triethylene glycol dimethacrylate (TEGDMA) resins (14 Wt%)	98420	VITA Zahnfabrik, Germany
Cerasmart (Cs)	Resin composite CAD/CAM blocks	Silica and barium glass nanoparticles (71 Wt%) in UDMA, DMA resin matrix.	1908226	GC Corp, Japan
Gradia Direct (Gr)	Direct microhybrid composite	Prepolymarized microfine ceramic fillers; silica, fluoro-alumino-silicate glass average silica, and fluoro-alumino-silicate particles (76 Wt%) in urethane dimethacrylate (UDMA).	2105101	GC Corp, Japan

**Table 2:** The mean and standard deviation values of flexural strength (MPa) of each material group under different storage media (n = 10). Different uppercase letters indicate significant differences in columns at set threshold of (*p* < 0.05) with actual significance level (*p* < 0.01) determined by one-way ANOVA. Different lowercase letters indicate a significant difference in lines at a set threshold of (*p* < 0.05) with actual significance levels (*p* < 0.0001) determined by of one-way ANOVA.

Storage Media	Gr	Cs	En
**Distilled water**	93.7 ± 12.5 ^aA^	236.4 ± 20.4 ^bA^	154.5 ± 15.7 ^cA^
**Gastric acid**	78.7 ± 8.6 ^aB^	246.6 ± 15.6 ^bA^	165.2 ± 9.8 ^cA^
**Coca-Cola**	86.3 ± 5.9 ^aAB^	232.9 ±15.6 ^bA^	162.2 ± 9.3 ^cA^

**Table 3:** The mean and standard deviation values of the elastic modulus (GPa) of each material group under different storage media (n = 10). Different uppercase letters indicate significant differences in columns at set threshold of (*p* < 0.05) one-way ANOVA. Different lowercase letters indicate a significant difference in lines at set threshold of (*p* < 0.05) with actual significance levels (*p* < 0.0001) determined by one-way ANOVA.

Storage Media	Gr	Cs	En
**Distilled water**	7.0 ± 0.8 ^aA^	10.3 ± 0.7 ^bA^	28.1 ± 1.2 ^cA^
**Gastric acid**	7.2 ± 0.8 ^aA^	11.0 ± 0.8 ^bA^	29.6 ± 1.7 ^cA^
**Coca-Cola**	7.2 ± 0.8 ^aA^	11.1 ± 1.1 ^bA^	29.1 ± 1.9 ^cA^

**Table 4:** The mean and standard deviation values of Vickers microhardness (VHN) before and after storage in gastric acid and Coca-Cola. Different uppercase letters indicate significant differences in columns (*p* < 0.05) using one-way ANOVA (*p* < 0.0001). Different lowercase letters indicate significant difference in lines (*p* < 0.05) according to a *t*-test (*p* < 0.0001). There was a significant effect of storage media on hardness reduction for all materials (superscript numbers) (*p* < 0.0001).

Storage Media	Gastric Acid			Coca-Cola		
Material	Before	After	Hardness Reduction %	Before	After	Hardness Reduction %
**Gr**	40.8 ± 0.4 ^aA^	28.3 ± 0.8 ^bA^	30.6% (1) ^1^	41.2 ± 2 ^aA^	34.6 ± 1.5 ^bA^	16% (0.25) ^2^
**Cs**	87.9 ± 1.2 ^aB^	80.3 ± 1.3 ^bB^	8.6% (0.08) ^1^	86.5 ± 1.6 ^aB^	80.2 ± 1.6 ^bB^	7.3% (00) ^2^
**En**	228.9 ± 0.8 ^aC^	205.3 ± 2.5 ^bC^	10.3% (2.1) ^1^	226.3 ± 2.7 ^aC^	212.1 ± 1.2 ^bC^	6.3% (0.22) ^2^

## Data Availability

The original contributions presented in the study are included in the article, further inquiries can be directed to the corresponding author.
